# Nanofibrous PCL-Based Human Trabecular Meshwork for Aqueous Humor Outflow Studies

**DOI:** 10.1021/acsbiomaterials.3c01071

**Published:** 2023-09-19

**Authors:** Maria Bikuna-Izagirre, Javier Aldazabal, Leire Extramiana, Javier Moreno-Montañés, Elena Carnero, Jacobo Paredes

**Affiliations:** † University of Navarra, TECNUN School of Engineering, Manuel Lardizabal 13, 20018 San Sebastián, Spain; ‡ University of Navarra, Biomedical Engineering Center, Campus Universitario, 31080 Pamplona, Spain; § Navarra Institute for Health Research, IdiSNA, C/Irunlarrea 3, 31008 Pamplona, Spain; ∥ Departamento de Oftalmología Clínica, Clínica Universidad de Navarra, Avenida Pio XII, 31080 Pamplona, Spain

**Keywords:** trabecular meshwork, electrospinning, polycaprolactone, outflow system, glaucoma, drug screening

## Abstract

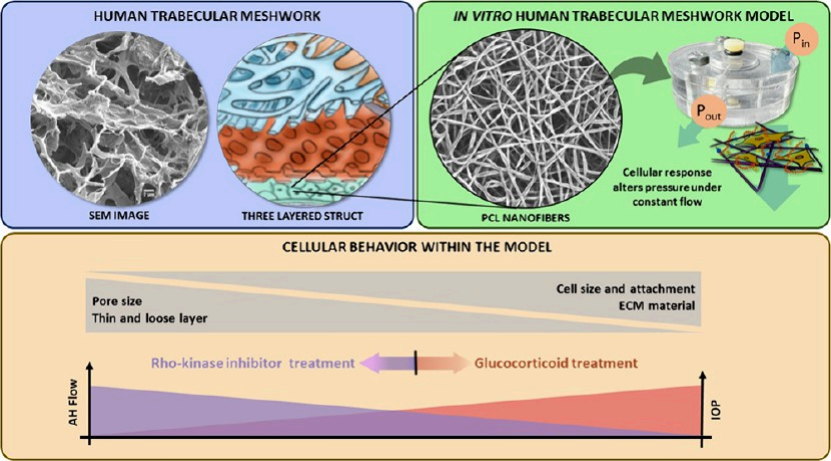

Primary open-angle glaucoma is characterized by the progressive degeneration of the optic nerve, with the high intraocular pressure (IOP) being one of the main risk factors. The human trabecular meshwork (HTM), specifically the juxtacanalicular tissue (JCT), is responsible for placing resistance to the aqueous humor (AH) outflow and the resulting IOP control. Currently, the lack of a proper *in vitro* JCT model and the complexity of three-dimensional models impede advances in understanding the relationship between AH outflow and HTM degeneration. Therefore, we design an *in vitro* JCT model using a polycaprolactone (PCL) nanofibrous scaffold, which supports cells to recapitulate the functional JCT morphology and allow the study of outflow physiology. Mechanical and morphological characterizations of the electrospun membranes were performed, and human trabecular meshwork cells were seeded over the scaffolds. The engineered JCT was characterized by scanning electron microscopy, quantitative real-time polymerase chain reaction, and immunochemistry assays staining HTM cell markers and proteins. A pressure-sensitive perfusion system was constructed and used for the investigation of the outflow facility of the polymeric scaffold treated with dexamethasone (a glucocorticoid) and netarsudil (a novel IOP lowering the rho inhibitor). Cells in the *in vitro* model exhibited an HTM-like morphology, expression of myocilin, fibronectin, and collagen IV, genetic expression, outflow characteristics, and drug responsiveness. Altogether, the present work develops an *in vitro* JCT model to better understand HTM cell biology and the relationship between the AH outflow and the HTM and allow further drug screening of pharmacological agents that affect the trabecular outflow facility.

## Introduction

**Figure 1:**
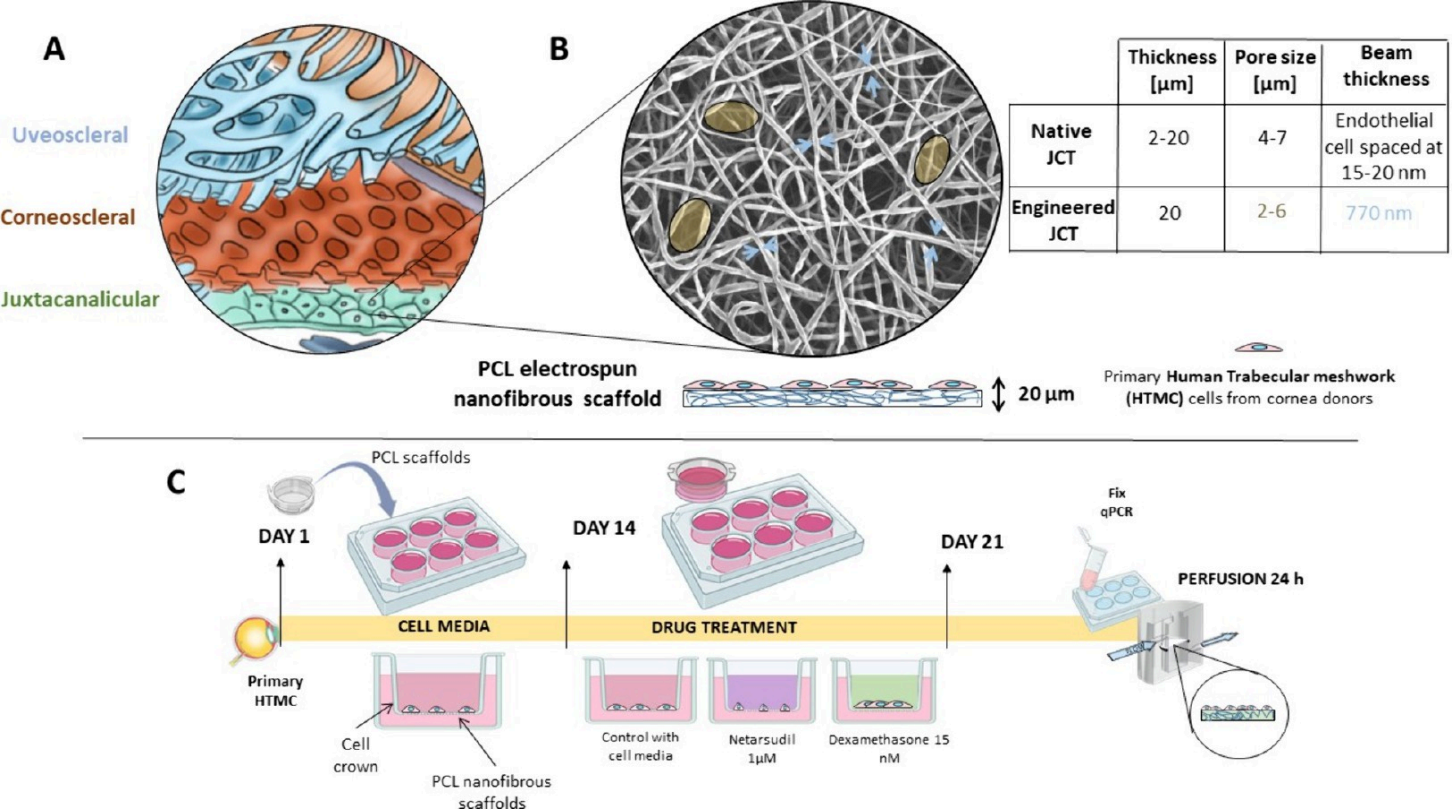
(A) Anatomical distribution of the HTM, formed by three layers: uveal, corneoscleral, and juxtacanalicular meshworks. (B) Scanning electron microscopy image of the engineered PCL electrospun nanofibrous scaffold indicating the morphological differences between the native and the engineered tissues. (C) Timeline of the experiments to validate the appropriateness of the polymeric scaffold.

Glaucoma is the leading cause of irreversible blindness worldwide, with a prevalence of 3.54% in the 40–80-year-old population.^1^ Primary open-angle glaucoma is the most predominant form of glaucoma, a complex neurodegenerative disease often associated with elevated intraocular pressure (IOP).^2^ Most of the currently available glaucoma therapies target aqueous humor (AH) production or the uveoscleral outflow pathway, which does not address the human trabecular meshwork (HTM) pathway (the conventional outflow pathway) responsible for 70–90% of AH drainage into the systemic circulation.^3−5^ Changes in physical properties of the HTM, such as the increase in stiffness (from 4 kPa in a healthy tissue to 80 kPa in a glaucomatous one),^6^ alterations in extracellular matrix (ECM) protein expression,^7^ or loss in its reparative capacity, can lead to increased outflow resistance, elevated IOP, and eventually glaucoma.^7,8^ Although pathogenetic mechanisms that lead to IOP elevation are still unclear, previous studies have identified the juxtacanalicular tissue (JCT) region, the inner part of the HTM after uveoscleral and corneoscleral layers, in conjunction to the inner wall region of Schlemm's canal as the primary site of outflow resistance due to the reduced pore size (0.5–2 μm) present in the JCT^9,10^ (Figure 1A).

A reconstruction of the JCT structure could help to understand how the AH outflow and the HTM are correlated in the natural environment and eventually lead to regenerative solutions. To address this need, different approaches can be found in the literature to try to imitate this native tissue. Human trabecular meshwork cell (HTMC) cultures in a microfabricated SU-8 scaffold with pores of 12 μm size were reported.^11−14^ The cells developed an HTM phenotype in terms of the morphology, expression of HTM cell-specific markers, and ECM secretion. The proposed 2D model although simple was able to mimic *in vivo* outflow physiology. Nevertheless, in this model, HTMC were only exposed to stiffer environments than the native tissue, and due to the overall construct thickness (<20 μm), they are not able to migrate into a 3D environment. In other studies, a functional 3D model was developed based on fibrillar hydrogels of collagen type I-elastin peptides, and the pathological state was successfully induced by dexamethasone treatment and counteracted by a rho-associated kinase (ROCK) inhibitor.^15^ Collagen and collagen/chondroitin sulfate scaffolds obtained via freeze-drying were also used to build 3D HTMC models. After 14 days of culture, HTMC were able to proliferate throughout the structure.^16^ On collagen, scaffolds containing glycosaminoglycans (GAGs) with different pore sizes (altered by a freeze-casting technique) indicated higher cell growth on larger pores, and fibronectin expression was upregulated with increasing GAGs and pore size, indicating the importance of the environment.^17^ Polyacrylamide gels of different rigidities have also been used to evaluate cell spreading and focal adhesions. They have shown that ECM rigidity modulates cytoskeletal structures, protein expression patterns, and fibronectin expression patterns in HTMC.^18^ A 3D culture using Matrigel^19,20^ showed promising results overcoming chronic oxidative stress, with improved results in dynamic conditions. Despite morphological and environmental modifications that hydrogels offer, their handling is not straightforward (polymerization rates, batch issues, fabrication of predesigned geometries, and lack of nutrient diffusion)^21^ and could hamper the repeatability of the experiments. Nevertheless, the particularity of the JCT tissue remains in the tight pores and the resistance that it offers to the AH outflow. A biomimetic JCT scaffold that could better reproduce its native morphological features and enable repeatable perfusion studies is still needed. The model presented in this research would help to closely understand the relationship between the AH outflow and HTM tissue.

In order to create an *in vitro* JCT model, the scaffolding material must be biocompatible and able to provide topographical cues to support HTMC growth and function. Polycaprolactone (PCL) has been widely used for cell culture and tissue engineering, and its desirable properties (biocompatibility, biodegradability, and mechanical characteristics) showed promising results mimicking the HTM.^22−24^ For instance, a micropatterned, ultrathin, porous PCL scaffold with different patterns showed the first steps toward an implantable HTM with proper HTMC growth, markers, and function.^25^ This technology offers high control in pore size and pattern design, offering thinner scaffolds than the native tissue though. Moreover, special facilities (clean room and qualified personnel) and more expensive and time-consuming processes are needed for this type of fabrication. Despite the novelty of this scaffold, *in vitro* perfusion studies and *in vivo* studies are still needed to validate the system as an implantable device, an objective far more challenging than our system.

Electrospinning technology enables tuning morphological and mechanical cues and maintaining equality between samples.^26^ A broad range of materials can be electrospun into nanofibrous membranes, including PCL. Recently, a novel 3D PCL-based scaffold for HTM cell culture was fabricated by melt electrowriting (MEW).^27^ This technology follows the same working principle as the solution electrospinning one, with custom-designed architectures and bigger fiber diameters though. The three-layered PCL-graded porous scaffold reported promising results mimicking the HTM. Cells were able to proliferate for 14 days with morphologies similar to those found in the native tissue. Although this scaffold is novel, no perfusion studies were performed. Moreover, MEW technology has some difficulties when creating small pores (due to electrostatic repulsive forces)^28^ and requires several stacked layers to increase the thickness of the final scaffold, hindering cellular infiltration.

Focusing just on the last layer of the HTM, the one offering most resistance to the AH outflow, the goal of this study is to validate a PCL-based electrospun scaffold as a JCT platform (Figure 1B) and evaluate the correct “physiological” behavior of HTMC in perfusion studies for drug screening purposes. To accomplish an *in vivo-*like physiology, the effects of cell seeding density, expression of HTMC markers, and secretion of ECM proteins followed by functional analysis of outflow characteristics using an *ad hoc* perfusion platform were evaluated (Figure 1C shows the timeline of the experiments). Finally, the bioengineered JCT model was treated with already well-documented drugs: the glucocorticoid dexamethasone (Dex), which is known for its glaucomatous effects (increase in ECM proteins and MYOC overexpression),^29,30^ and the rho inhibitor netarsudil (Net) (AR-13324), which has shown successful results in reducing the IOP in human studies due to the loss of focal adhesions and the cytoskeleton disruption.^31,32^ Thus, our aim is to address whether the system responds to these drugs as described in the literature and mimics the *in vivo* outflow physiology.

## Materials

All chemicals were purchased from Sigma-Aldrich (St. Louis, MO) unless otherwise specified.

## Methods

### Human Trabecular Meshwork Cell Culture

Human donor eyes were obtained from the Center for Medical Research and Education (Universidad de Navarra, Spain). In total, 23 HTM donors (12 women and 13 men) with an average age of 71 ± 15.1 were obtained. Primary HTMC were isolated as indicated by Du et. al.^33^ Cells from two donors were used for the following experiments (patient medical histories are not available). The HTMC were initially seeded on 0.1% gelatin-coated 75 cm^2^ cell culture flasks and cultured in high-glucose DMEM with 10% fetal bovine serum (FBS). Fresh culture media were supplied every 48 h. Cells were maintained at 37 °C in a humidified atmosphere with 5% carbon dioxide until 80–90% confluence was reached at which point cells were trypsinized using 0.05% trypsin EDTA (Gibco, Thermo Fisher, Waltham, MA, USA) and subcultured. All studies were conducted using cells before the fourth passage.

### Nanofibrous Scaffold Fabrication

First, PCL (*M*_n_ of 80,000) (10 wt %) was dissolved in the chloroform/methanol (Panreac, Barcelona, Spain) solvent mixture with a volume rate of 4:1 v/v with agitating the mixture with a magnetic stirrer at 600 rpm overnight at room temperature (21 ± 1 °C).

Nanofibers were fabricated on an upright custom-made setup (Figure S1) using a controlled flow rate (0.5 mL/h) with a syringe pump (Chemyx Fusion 100, Texas, USA), which was connected to a blunt metallic needle of 20G through a capillary Teflon tube. A high-voltage DC power supply (FC series 120 W, CE compliant) was used to provide the necessary electric field between the needle and the collector.

For the process of electrospinning, PCL (10 wt %) was placed in a 1 mL plastic syringe, and a voltage of 13 kV was applied, with a distance of 10 cm between the needle and the collector. The nanofibers (Figure S3) were collected on a 9 × 9 cm^2^ flat aluminum foil.

### Characterization of the PCL Nanofibrous Scaffold

The morphology of the nanofibrous scaffolds was studied with field-emission scanning electron microscopy (SEM) (Zeiss, Gemini, Germany) at an accelerating voltage of 5 kV. Fiber diameters and pore areas of the scaffolds were calculated on the basis of SEM images by using image analysis software (ImageJ, NIH, USA) and DiameterJ plugin (Nathan Hotaling–v1.018), respectively.

Mechanical measurements were obtained by applying tensile test loads to the electrospun nanofibrous mats. The specimens were cut 11 mm in width and placed on a traction testing machine (ZwickLine Z1.0, Zwick/Roell, Germany) with a load cell of 50 N (XForce P, Zwick/Roell, Germany) at room temperature. The initial distance between grips was 10 mm (the studied area was 11 × 10 mm^2^), and the crosshead displacement speed was set to 100 mm/min for all tests. The thickness was measured with a thickness digital gauge (Digimatic series 547, Mitutoyo).

### Evaluating HTMC on PCL Scaffolds

PCL (10 wt %) scaffolds were treated with a plasma (100 W for 1 min, with 5 and 15 sccm of O_2_ and Ar, respectively) process (Diener Electronic, Germany) in order to increase hydrophilicity of the surface. Then, electrospun meshes were placed on 24-well plate cell crowns (Scaffdex, Finland) and were sterilized with UV radiation from both sides for 30 min each. Subsequently, the scaffolds were coated with 0.1% gelatin for 30 min, and HTMC were seeded on the scaffold at various cell densities (2.5 × 10^4^, 5 × 10^4^, 10 × 10^4^, 20 × 10^4^, and 40 × 10^4^ cells per well) for 7 days. After the incubation period, 50 μL of 3-(4,5-dimethylthiazol-2-yl-2,5-diphenyltetrazolium bromide) (MTT) from Roche was added to each well. The media was discarded, and 400 μL of DMSO was added to the wells overnight at 37 °C. Finally, the optical density (OD) value was measured at 490 nm using a spectrophotometer.

### HTMC Response to Drugs on PCL Scaffolds

The 10% PCL scaffolds after being sterilized and coated with 0.1% gelatin were seeded at 10 × 10^4^ cells/well and grown for 14 days. After this incubation period, dexamethasone at 15 nM^34^ and netarsudil hydrochloride (CymitQuimica S.L., Barcelona) at 1 μM^35^ were added for 5 days. Samples were collected for the study of gene expression by a real-time polymeric chain reaction (qPCR).

### Scanning Electron Microscopy (SEM)

The cell morphology was characterized using SEM, 14 days after cultures were initiated. For SEM imaging, samples were fixed with 4% paraformaldehyde (PFA) for 1 h at room temperature. The samples were rinsed three times in Dulbecco's phosphate-buffered saline (DPBS), dehydrated in a graded ethanol series (50, 70, 80, 95, and 100%) for 5 min each at room temperature, and air-dried. Fixed samples were mounted on stubs and sputter-coated with palladium at 18 mA for 75 s. Images were obtained at an accelerating voltage of 5 kV.

### Inmunochemistry and Confocal Imaging

Cells were fixed and stained for the F-actin cytoskeleton, focal adhesions, ECM proteins, and the nucleus. After 14 days in culture, the cells grown on PCL scaffolds were fixed with 4% paraformaldehyde, permeabilized with 0.3% Triton X-100, and blocked with 2% bovine serum albumin in PBS. HTM cells were subsequently incubated with primary antibodies (mouse antivinculin (Proteintech, UK), 1:300; mouse antimyocilin (Abcam), 1:200; rabbit anticollagen IV (Abcam), 1:400; rabbit antifibronectin (Sigma), 1:200; mouse anti-β-catenin (BD), 1:250) overnight. Next, the secondary antibodies (goat antimouse cy3 (1:500) for vinculin, phalloidin Alexa Fluor 488 (1:200) for F-actin fibers, donkey antirabbit 488 (1:1000) for myocilin and collagen, donkey antirabbit 555 (1:1000) for fibronectin, and donkey antimouse 555 (1:1000) for β-catenin) were applied overnight at 4 °C. Finally, 4′, 6-diamidino-2-phenylindole (DAPI) (1:500) was used to stain cell nuclei.

Laser scanning confocal microscopy was performed using a Zeiss LSM 980 confocal microscope (Zeiss, Germany), and images were acquired at 10× magnification. Confocal images were processed using Zen software, and all confocal images within a given experiment were captured using the same laser intensity and gain settings in order to compare intensities across samples. The mean fluorescence intensity (MFI) of vinculin, myocilin, collagen IV, β-catenin, fibronectin, and F-actin was determined using *Z*-project maximum intensity projections in ImageJ with image background subtraction.

### Perfusion Studies

A controlled flow system circuit for pressure measurements was developed, as shown in Figure S2, combined with a custom-designed perfusion chamber with integrated pressure sensors (Elveflow, Paris, France). This flow system allowed for simultaneous control of flow and measurement of the transmembrane pressure, enabling study of the outflow characteristics in our *in vitro* HTM model. Fourteen days prior to perfusion measurements, a concentration of 10 × 10^4^ HTMC per well was seeded on 10% PCL scaffolds (grown in cell crowns). At day 21, PCL scaffolds with a confluent layer of HTMC were secured in the camera and perfused at 10, 20, 40, 80, and 160 μL/min for 24 h, with a perfusion medium consisting of DMEM with 1% l-glutamine and 1% penicillin-streptomycin. The perfusion chamber was introduced in the incubator at 37 °C. The pressure difference at the beginning and end of the circuit was permanently monitored with pressure sensors and recorded.

After 14 days of culture, HTMC were under these chemicals for 7 days more, in 6-well culture plates. Subsequently, each PCL scaffold was transferred to an individual perfusion camera, setting up the circuit with three chambers (control, Dex, and Net) and two pressure sensors for each. Samples were perfused with 15 nM Dex and 1 μM Net both dissolved in 10% DMEM in FBS at the same constant flow rate (20 μL/min) for 24 h. Control samples were perfused with the cell media. Samples were fixed for SEM, staining, and confocal image analysis as described above. Figure 1C graphically explains the time sequence of the experiment.

The outflow facility or hydraulic conductivity is the ratio of the flow rate (*F*) to the change in pressure (*P*) (Δ*F*/Δ*P*) and measures how easily a fluid can pass through a membrane. High values indicate lower outflow resistance. First, the slope of the *P* vs *F* graph was calculated, and then, the outflow facility was determined from the inverse of the slope per unit area. Four different samples per condition were studied under perfusion. Normalization of the outflow facility to the surface area was calculated as indicated on the work of Torrejon et al.^13^

### Quantitative Real-Time PCR (qPCR) Analysis

Total RNA was extracted from samples cultured for 21 days with or without treatment (Dex and Net) using a Maxwell kit as per the manufacturer's instructions (Promega, Madrid, Spain). RNA was quantified by using a NanoDrop spectrophotometer (Thermo Scientific, Wilmington, DE). PCR probes and primers were generated using Primer3Plus (https://www.bioinformatics.nl/cgi-bin/primer3plus/primer3plus.cgi) design software. Specific primer sequences are given in Table 1. Samples were amplified using an SYBR green I PCR master mix. Reactions were analyzed in triplicate, and expression levels were normalized to the housekeeping GAPDH. Relative quantitative data analysis was performed using the Livak method (2^**–(ΔΔCt)**^). Experiments were performed in triplicate, and the average values are presented as means ± standard deviation (SD).

**Table 1:** The Sequence of the Primer Set Used in the Study

**name**	**forward**	**reverse**
***ACTA2***	5′-CTG ACC CTG AAG TAC CCG AT-3′	5′-GTC ATT TTC TCC CGG TTG GC-3′
***FN1***	5′-CAT GAA GGG GGT CAG TCC TA-3′	5′-CCT TCT CCC AGG CAA GTA CA-3′
***ITGβ3***	5′-AGA TGC GAA AGC TCA CCA GT-3′	5′-TCC GTG ACA CAC TCT GCT TC-3′
***MMP9***	5′-GAG ACC GGT GAG CTG GAT AG-3′	5′-TAC ACG CGA GTG AAG GTG AG-3′
***MYOC***	5′-AGT TCC TGC TTC CCG AAT TT-3′	5′-CTC GCA TCC ACA CAC CAT AC-3′
***VIM***	5′-TGC CTC TTC CAA ACT TTT CCT C-3′	5′-CGT GAT GCT GAG AAG TTT CGT-3′
***GAPDH***	5′-TGG AAG GAC TCA TGA CCA CA-3′	5′-TTC AGC TCA GGG ATG ACC TT-3′

### Statistical Analysis

Data were expressed as the average ± SD. In the perfusion studies, the difference between controls and Dex-treated and Net-treated nanofibrous PCL HTM samples was analyzed using one-way or two-way ANOVA. GraphPad Prism software v9.3 (GraphPad Software, La Jolla, CA, USA) was used for all analysis (*p-value < 0.05, **p-value < 0.01, ***p-value < 0.001, and ****p-value < 0.0001).

## Results

### Characterization of the PCL Nanofibrous Scaffold

To recapitulate this tiny porous structure and outflow characteristics of the HTM, we fabricated PCL nanofibrous scaffolds. These nanofibrous scaffolds (Figure S3) have fiber diameters of 770 ± 0.172 nm and pore sizes of 5.59 ± 0.28 μm^2^. The pore sizes in these scaffolds were obtained thanks to the small fiber dimensions, closely mimicking the JCT structure and as they are smaller than the size of a single HTMC, enabling cell spreading. A previous study has observed that HTMC have difficulty growing into a confluent layer on larger pore size structures and are unable to expand over the pores.^11^ The thickness of these freestanding scaffolds was measured to be 20.25 ± 1.25 μm through a digital gauge, and the elastic modulus was 0.95 ± 0.05 MPa, close to values reported in the literature,^27^ greater than the native trabecular meshwork though (4 kPa).^6^

### Evaluation of PCL-Based HTM Scaffolds as a Cell Culture System

**Figure 2:**
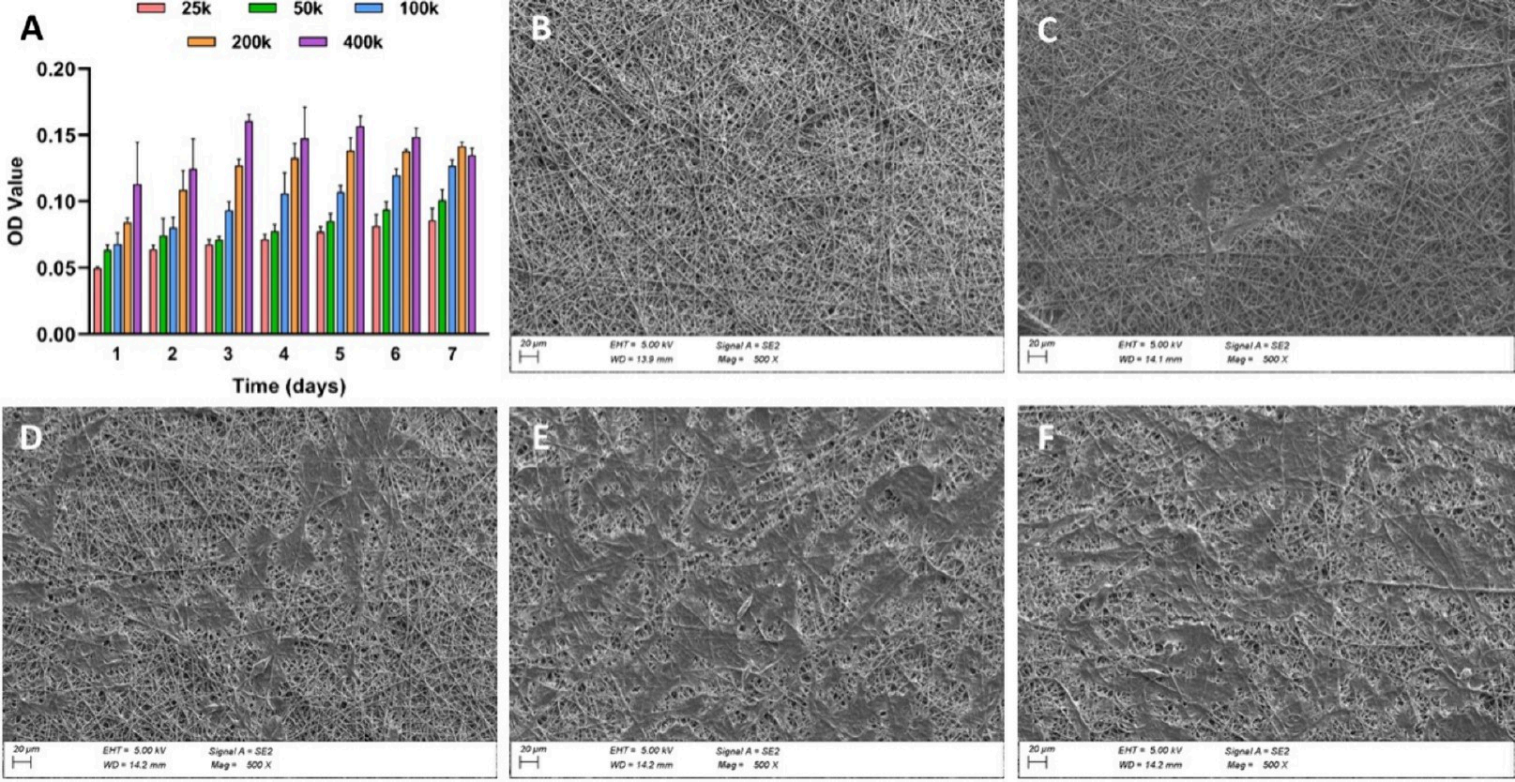
(A) Bar plot of MTT results with different HTMC initial seeding concentrations on the PCL 10% nanofibrous scaffold after 7 days. SEM images of HTMC after 7 days with initial concentrations of (B) 2.5 × 10^4^, (C) 5 × 10^4^, (D) 10 × 10^4^, (E) 20 × 10^4^, and (F) 40 × 10 ^4^ cells/well. Scale bar: 20 μm.

The initial seeding density (2.5 × 10^4^, 5 × 10^4^, 10 × 10^4^, 20 × 10^4^, and 40 × 10^4^) on HTMC attachment and growth on PCL scaffolds was evaluated by an MTT assay for 7 days (Figure 2A). The lowest concentrations (2.5 × 10^4^ and 5 × 10^4^) did not show enough cell spreading after 7 days of culture as presented in Figure 2B,C and so were discarded as initial seeding concentrations. From Figure 2D, an initial monolayer formation can be observed without reaching a whole coverage. The bar plot in Figure 2A indicates faster cellular proliferation at 20 × 10^4^ and 40 × 10^4^ cell concentrations. Indeed, 40 × 10^4^ cells/well reached the peak by the 3rd–4th days, reaching a constant value to end up decreasing by day 7. Similarly, 20 × 10^4^ showed a similar pattern that shifted by 1–2 days, reaching a plateau state by the end of the experiment. In both cases, SEM images show a monolayer formation with little room for further proliferation (56 and 45%, respectively) (Figure 2E,F). Finally, the concentration of 10 × 10^4^ cells/well showed a progressive increase without reaching the peak by day 7 (data related to area coverage analysis are shown in Figure S4).

### Biological Characterization of the PCL-Based HTM Scaffold

**Figure 3:**
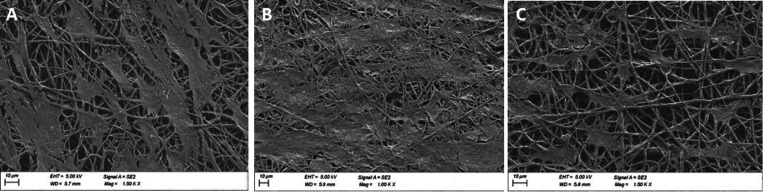
SEM images for primary HTMC morphology evaluation in PCL nanofibers of (A) control, (B) dexamethasone, and (C) netarsudil. Scale bar: 10 μm.

Once we verified the suitability of the electrospun scaffolds to culture HTMC, we confirmed HTMC-specific gene expression by qPCR analysis and morphological changes by immunochemistry and SEM imaging. Based on the SEM images shown in Figure 3, evident morphological alterations can be observed. The cellular coverage percentage indicated that control samples covered 43.49 ± 6.46 of the images, Dex-treated samples the 84.16 ± 11.38 indicating an increase in cell size, and Net-treated ones the 22.10 ± 2.86 with a reduction in the area covered by cells provoked by the inhibition of focal adhesions and actin microfilament disruption. The statistical difference was found among the three groups (*p* < 0.05) (Figure S6).

**Figure 4:**
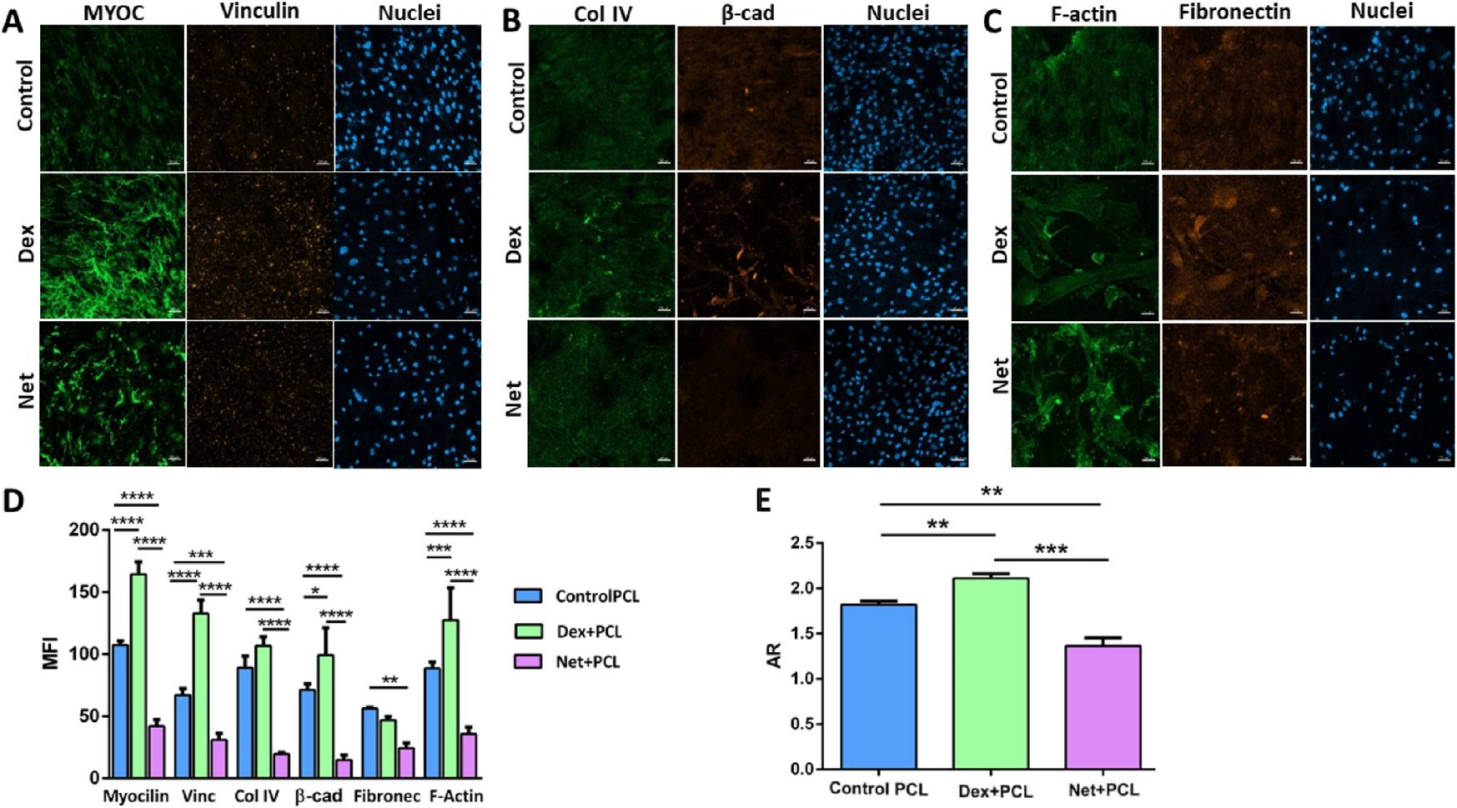
Confocal images of HTMC after 21 days of culture: (A) myocilin and vinculin, (B) collagen IV and β-catenin, and (C) F-actin fibers and fibronectin. (D) Quantification of confocal images and mean fluorescence intensity (MFI) and (E) nucleus aspect ratio.

The HTMC marker myocilin was stained in the control, Dex, and Net groups. Figure 4A shows an increase in myocilin expression for the Dex samples and a decrease in the Net group. Other ECM proteins, like collagen IV (Figure 4B) and fibronectin (Figure 4C), showed an increase in their expression when facing Dex treatment, not in Net cases though where the MFI of these proteins was shown to be lowered (Figure 4D). The actin cytoskeleton, with a filamentous F-actin fiber arrangement, directly affects cell and tissue contraction. F-actin fibers are involved in HTMC contractility regulation and is a therapeutic target for lowering IOP. As expected, Net caused disassembly of the stress fibers, and diffuse green patches of intracellular staining appeared (Figure 4C).^35,36^

The nucleus shape in cells treated with different drugs based on DAPI was estimated after 21 days of culture. The aspect ratio (AR) parameter was calculated as a ratio of the nucleus length to width (example provided in Figure S7). The nuclei of cells were elongated (AR > 1.5) in control samples, with an AR of 1.82 ± 0.12, typical in he native HTM^11^ (Figure 4A–C). Dex treatment reported an increase in cell nucleus size (AR = 2.07 ± 0.07) as a consequence of glucocorticoid-induced alterations in these cells.^37^ Net-treated samples instead showed a more rounded nucleus shape with a reduction in their AR = 1.36 ± 0.22 due to the effects of rho inhibitor-based treatment^38^ (AR values are shown in Figure 4E). The ability of cells to change their nucleus morphology indicated that the PCL nanofibrous scaffold enables correct cellular behavior.

**Figure 5:**
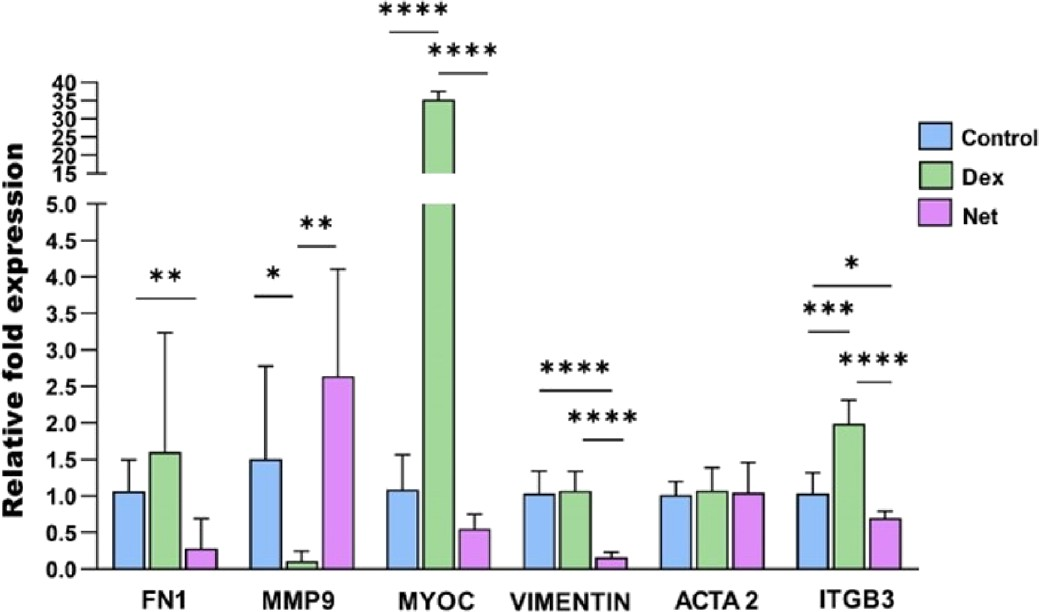
qPCR analysis of gene expression of ACTA2, FN1, ITGβ3, MMP9, MYOC, and VIMENTIN after dexamethasone (15 nM) and netarsudil (1 μM) treatment.

These changes in the cell morphology and ECM proteins were echoed in gene expression analysis shown in Figure 5. HTMC exposed to 7 days treatment with Dex significantly increased expression of MYOC (34.04-fold) and ITGβ3 (2.06-fold), while MMP9 was almost undetectable, and no significant results were obtained for ACTA2, FN1, and VIMENTIN when compared to the control. Low values for MMP9 indicated a reduction in ECM remodeling (low collagen IV degradation), observing higher MFI values in the confocal images (Figure 4B,D). Net-treated samples showed a significant decrease in these genes VIMENTIN (0.12-fold), ITGβ3 (0.67-fold), and FN1 (0.59-fold) compared to the control. The downregulation of focal adhesions provoked the disruption of F-actin fibers (Figure 4C), which hindered the contraction force. Moreover, a reduction in ECM protein expression was also observed with an increase in collagen IV degradation (caused by MMP9 overexpression; Figure 4B) and a reduction in fibronectin (FN1 downregulation; Figure 4C).

Note that in Figure 4C, the cell density varies between groups. This was because images were taken around the edge of the scaffold instead of the middle. PCL nanofibers provide such a background noise that the difference in F-actin fibers was not detectable. Thus, we moved into the edges, where the fiber density decreased, and clearer images were possible to take. Figure S8 shows different confocal images taken in the middle of the scaffold.

### Outflow Studies of the Electrospun HTM

**Figure 6:**
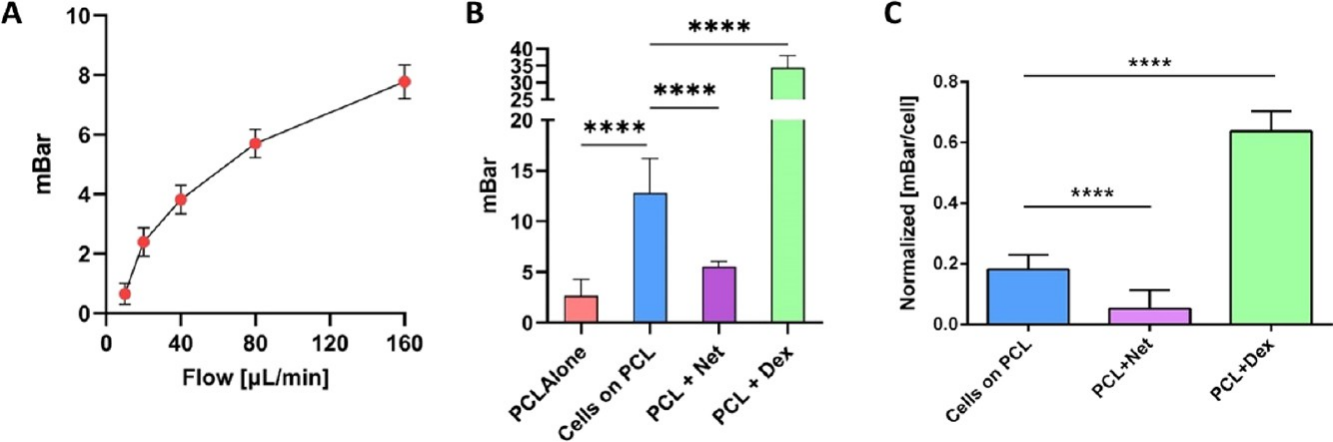
Outflow studies of the bioengineered HTM on PCL nanofibrous scaffolds. (A) Determination of the outflow facility of the artificial HTM through the relationship of the transmembrane pressure and flow rate under perfusion. (B) Resistance difference to flow in the PCL scaffold with and without HTMC, after dexamethasone (15 nM) and netarsudil (1 μM) treatment. (C) Normalized pressure data to the number of alive cells in each group.

The fact that HTMC grown on PCL nanofibrous scaffolds maintained an HTMC-like phenotype prompted us to further evaluate the outflow facility of the electrospun HTM using a perfusion system, as shown in Figure S2. HTMC cultured on the PCL scaffold for 14 days were incorporated into a watertight chamber, where the pressure across the tissue construct was measured maintaining a constant flow rate (20 μL/min). HTMC provided flow resistance, raising the transmembrane pressure to 12.79 ± 0.62 mbar, while PCL scaffolds alone (without HTMC) had no significant resistance to flow (transmembrane pressure of 2.39 ± 0.43 mbar). Pressure measurements at different flow rates (10, 20, 40, 80, and 160 μL/min) allowed for calculation of the outflow facility of the electrospun scaffold with no cells. The slope of the transmembrane pressure (*P*) versus flow rate (*F*) curve was 0.047 ± 0.02 μL/min/mbar (Figure 6A), and the outflow facility (Δ*F*/Δ*P*), calculated from the inverse of the slope, was found to be 21.27 ± 0.02 μL/min/mbar.

### Physiological Response of the Electrospun HTM to Dexamethasone and Netarsudil

One of the main functions of the HTM is to regulate the AH outflow. To further evaluate whether our system allows HTMC to behave in a “physiological manner”, Dex (15 nM) and Net (1 μM) were added to the perfusate for 24 h.

**Figure 7:**
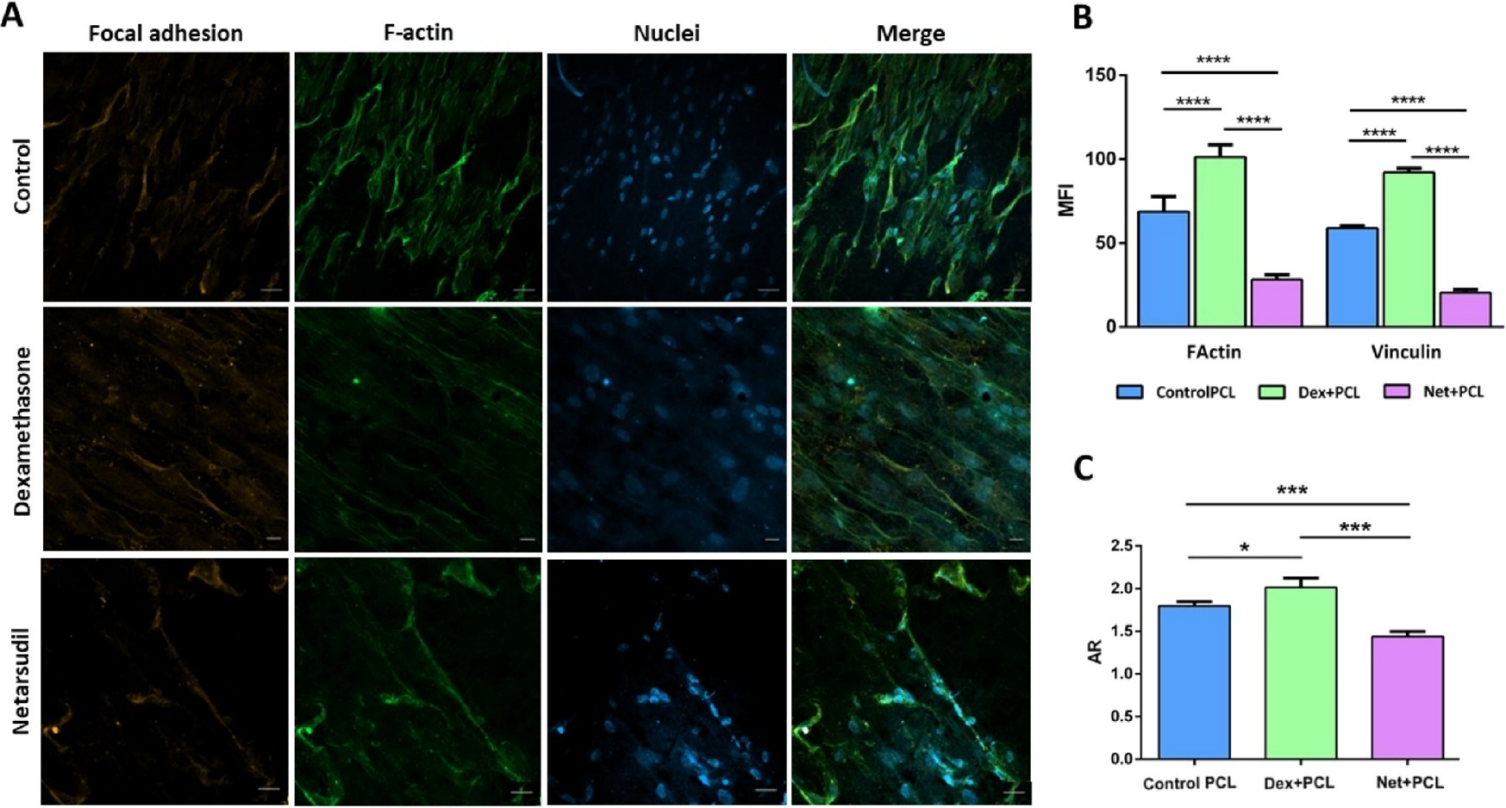
(A) Confocal images of HTMC on PCL scaffolds after perfusion with 15 nM dexamethasone and 1 μM netarsudil. The first column indicates the focal adhesions (vinculin), the second column the F-actin fibers (phalloidin Alexa Fluor 488), the third column the nuclei (DAPI), and the last column the merged images. (B) Mean fluorescence intensity (MFI) of confocal images and (C) nucleus aspect ratio.

**Figure 8:**
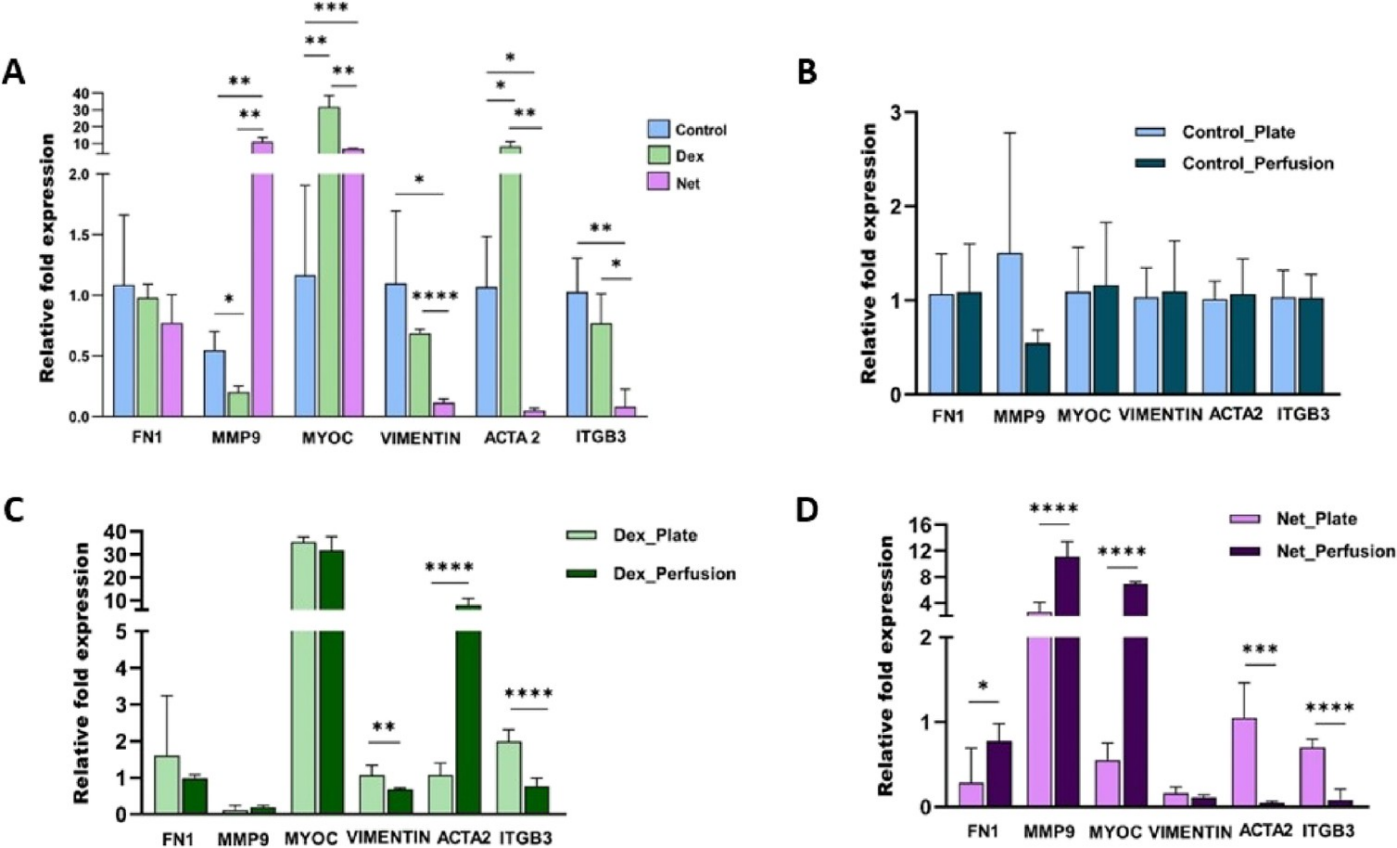
qPCR analysis of (A) ACTA2, FN1, ITGβ3, MMP9, MYOC, and VIMENTIN expression after perfusion studies with 15 nM dexamethasone and 1 μM netarsudil. Comparing the qPCR, results between culture plates and perfusate samples: (B) control samples, (C) dexamethasone, and (D) netarsudil.

Regarding Dex perfusion results, the transmembrane pressure of the PCL-based HTM increased dramatically from 12.79 ± 0.62 to 34.44 ± 1.72 mbar after Dex perfusion, indicating increased flow resistance (Figure 6B,C). This pharmacological agent increased resistance to flow by 169 ± 7% (*N* = 4, *p* < 0.05). Furthermore, Dex appeared to decrease the outflow facility of our system by inducing an increase in the number of F-actin fibers and secretion of ECM proteins. Confocal images showed that focal adhesion together with F-actin fibers was boosted (Figure 7A,B), suggesting that an increase in the size of the cells and in the nucleus AR (Figure 7C), and the amount of ECM material increased resistance to outflow in our system. These results were confirmed by qPCR (Figure 8A), which showed results similar to those previously exposed. Dex treatment causes an overexpression in cytoskeletal production (MYOC and ACTA2 increase) causing the overproduction of the cytoskeleton structure, focal adhesions (ITGβ3), and ECM proteins (FN1 and MMP9).

Results for Net perfusate samples match the literature. Net is a potent ROCK inhibitor, which inhibits rho kinase (induces disassembly of actin stress fibers) and norepinephrine transporters found in the HTM pathway, decreasing AH outflow resistance and lowering IOP.^31,35,39^ The transmembrane pressure decreased by 57.4 ± 3% (*N* = 4, *p* < 0.05) from 12.79 ± 0.62 to 5.45 ± 0.39 mbar (Figure 6B,C). Additionally, Net appeared to increase the outflow facility of our system by minimizing the focal adhesions, thus causing cytoskeleton disruption and reducing the nucleus AR (Figures 7A,C and 8A). The confocal images showed that actin fibers were disturbed and cells have lost their elongated spindle-shape appearance, suggesting the critical role of actin filaments in maintaining the HTMC morphology and outflow physiology.

To explore the effect that the flow may exert on HTMC, the qPCR results of a 21-day course study of well plates and perfused ones were compared (Figure 8B–D). Interestingly, control samples did not show any statistically significant results (Figure 8B) when the flow was present. In treated samples though, both Dex (Figure 8C) and Net (Figure 8D) showed a reduction in focal adhesions (ITGβ3 and VIMENTIN) and an increase in MMP9 (especially in the Net case (*p* < 0.0001)). Figure 8C shows how in the Dex case the flow exacerbated ACTA2 expression, whereas in the Net group, its expression was negligible (Figure 8D). Somehow, the flow induced more changes in the presence of Net, where an increase in FN1 and MYOC was also observed. Overall, these data demonstrate a complex ECM regulation that takes place as a result of having a constant flow.

## Discussion

In this study, we investigated the feasibility of using electrospun PCL nanofibrous scaffolds to support HTMC growth into an *in vitro* HTM model system for outflow studies. Conventional HTM cell culture on plastic tissue-culture surfaces is useful for fundamental studies of HTM biology, and it cannot be used for outflow physiology studies.^40^ One of the main referent in HTM engineered models is the SU-8-based outflow platform, which has allowed perfusion studies with different drugs.^11−14^ However, HTMC were not able to migrate into a 3D environment and were influenced by surface morphologies and stiffness. Hydrogels have also shown their suitability for HTMC behavioral studies,^16,17^ especially in morphological and stiffness-related cues.^20,41,42^ Electrospinning-based PCL scaffolds have also been used to imitate the three-layered graded porous HTM architecture. Despite the promising results, MEW requires specialized machines, and its scaffolds present limitations when creating small pores.^27^ As we focused on the creation of an artificial JCT, we propose a PCL-based scaffold based on the solution electrospinning technique.^22^ This technology permits an easy manipulation of the fabrication parameters,^43^ where mechanical properties and pore sizes can be modified in the membranes achieved.^44^

Based on the information that we have gathered about the HTM, the JCT is the tissue offering the most resistance to the AH outflow. Formed by a connective tissue and made up of a continuous layer of endothelial cells spaced at 15 to 20 nm and pores 0.5 to 2 μm in diameter^10^ (Figure 1B), we created a PCL-based scaffold with a pore size of 5.59 ± 0.28 μm^2^ and a fiber diameter of 770 ± 0.172 nm (Figure S3), which closely match the JCT characteristics. Mechanical characteristics though are far stiffer than the native tissue (0.95 ± 0.05 MPa against 4 kPa), which are assigned by the polymer and the fabrication parameters. Electrospinning in general will provide a quite high elastic modulus (around MPa or beyond), as it is based on polymer usage.^22−24^

An essential part of this work was to validate the nanofibrous polymeric scaffold as an *in vitro* JCT model to perform AH outflow studies. Hence, we have evaluated the responsiveness of HTMC on the PCL-based scaffold when treated with two well-documented drugs: Dex^45,46^ and Net.^31,35,39^ The studies performed in well plates presented the expected results with an increase in cell size and area coverage (84.16 ± 11.38%) when treated with Dex and a noticeable decrease in the Net surface (22.10 ± 2.86%) compared to the control samples (43.49 ± 6.46%) (Figure 3 and Figure S6). These morphological alterations were supported by confocal images (Figure 4A–C) and qPCR results (Figure 5). As reported before, Dex treatment increases the myocilin expression (Figure 4A) (MYOC gene (Figure 5)), an HTMC marker whose overexpression is related to glaucoma cases.^45,46^ Moreover, an increase in ECM proteins, F-actin fibers, and focal adhesions was also observed as expected, with higher MFI levels in collagen IV, fibronectin, vimentin, F-actin fiber, and β-catenin signals. On the contrary, with Net being a potent rho inhibitor, HTMC have a disrupted morphology due to the loss of focal adhesion and the consequent cytoskeleton disorganization. As shown in previous studies, confocal images showed a reduction in focal adhesions, ECM protein expression, and F-actin fibers (Figure 4A–C), which were endorsed by qPCR results (Figure 5). These results validate an adequate HTMC behavior when facing different drugs after 21 days of culture on the *in vitro* JCT model.

Considering the results in the nanofibrous scaffolds, perfusion studies were performed in a custom-made perfusion platform (Figure S2). This system showed to be able to measure pressure differences caused by the morphological changes that the HTMC suffered after drug treatment. Indeed, due to the increase in ECM proteins, F-actin fibers, and thus in cell size, Dex treatment significantly increased the pressure difference within the system (169 ± 7%) compared to control samples (Figure 6B,C). Again, confocal images and qPCR results supported these values (Figures 7A and 8A) with MYOC (31.90-fold), ITGβ3 (0.77-fold), and ACTA2 (8.15-fold) overexpression. Similarly, the Net case showed the expected results with a decrease in transmembrane pressure (57.4 ± 3% reduction) as a consequence of F-actin fiber disruption and following morphological loss (Figure 7A). The fact of introducing a constant flow may cause alterations in the genetic expression of HTMC in the presence of drugs. Control samples indeed did not present any statistically significance when comparing qPCR results of well plate and perfused samples (Figure 8B). However, we saw an interesting reduction in focal adhesions (ITGβ3 and VIMENTIN expressions) in both Dex and Net groups. This may indicate that the flow may exert an external force, which alters the cell adhesion. Moreover, we observed some statistically significant results in the Net case, with a reduction in collagen IV degradation and an increase in fibronectin and MYOC expression. In Dex though, fibronectin and MYOC were slightly downregulated compared with well plate results. Despite these results being interesting, we can affirm only that the presence of a constant flow may cause a complex ECM alteration and that of course, this notion is beyond our knowledge as well as out of the scope of the main objective of the study.

Our results are consistent with previous *in vitro* studies, including perfusion studies using Dex, where the mean IOP increase of 23 mbar, the thickening of beams,^47^ and increased overall acting staining (cytoskeletal changes) were reported.^48,49^ In perfusion studies using Net, similar results to ours were shown, with a significant decrease in outflow resistance (51%)^32^ and reduction in ECM accumulation.^12^ These results confirm that our nanofibrous HTM model is responsive to IOP (increasing/lowering) drugs and can be used to study aqueous outflow *in vitro*. The perfusion system implemented in this work allowed for calculation of the outflow facility of the artificial JCT. The outflow facility provides insight into the ability of the polymeric HTM to resist flow under pressure. In our system, this resistance is created by the HTMC themselves; the PCL nanofibrous scaffold alone did not significantly resist flow. The outflow facility calculated in our experiments is considerably higher than the native HTM^50^ (21.27 μL/min/mbar (28.05 μL/min/mmHg) compared to 0.271 μL/min/mmHg *in vivo*); this discrepancy was expected. The native HTM has a high level of complexity with multiple different layers, a significant number of cells, and ECM material in addition to a different sectional area, which as a whole contributes to a lower outflow facility. It is well-known that a large part of outflow resistance *in vivo* is provided by the ECM of the JCT and the inner wall of SC.^7,51^ Although the value of the outflow facility of the current nanofibrous JCT model is higher than that *in vivo*, it provides a valuable outflow system that may serve as a high-throughput platform for IOP drug screening. We anticipate further manipulation of the properties of scaffolds, increasing deposition time (to create a thicker mesh), changing the material (stiffness alterations), or combining solution electrospinning with MEW. These uploads will help in achieving an upload to the current scaffold, moving from one-layered model (JCT) to an improved artificial HTM (three-layered scaffold) that better reflects the architecture and physiology and thus provides an outflow facility value closer to the one measured in a native tissue.

We have successfully fabricated electrospun PCL nanofibers to support HTMC and their correct behavior. Although our results were consistent with those found in the literature, our scaffold faces some limitations. For instance, it provides a higher elastic modulus than the native tissue, which may alter cellular performance. As reported by several groups, the stiffness of the substrate is becoming a key factor^18,52^ in the glaucoma field and is becoming an important research line. Fortunately, electrospinning enables tuning the stiffness of the substrate, altering the elastic moduli of the scaffold (changing the polymer, a combination of synthetic and natural polymers), which makes this platform promising for these studies. Moreover, our model presents small pore sizes, which allow an easy monolayer formation but limit cellular infiltration to the outermost layers of the scaffolds (around 5 μm (Figure S5)); also, plasma treatment and gelatin coating are needed to promote cell adhesion. Nevertheless, our JCT *in vitro* model could go a step beyond and be combined with MEW technology to create the corneoscleral and uveoscleral layers to better recapitulate the three-layered architecture of the native HTM. Overall, this work showed a successful attempt to mimic the JCT, based on a PCL nanofibrous scaffold. In this study, we have validated our engineered JCT (showing correct HTMC growth and response) and custom-designed perfusion platform, which measured the pressure differences caused by HTMC when facing Dex and Net. Regarding the perfusion platform, attempts for long-lasting perfusion studies will be sought (from 24 h to several days) as well as pressure-dependent studies, in order to research pressure effects on HTMC or retinal ganglion cells. This development pretends to help in the understanding of key biological and physiological cues related to glaucoma and AH outflow. In that way, new targets could be identified, leading to more effective therapies.

## Conclusions

This study confirms the feasibility of using solution electrospinning PCL-based scaffolds to construct an *in vitro* JCT model for investigation of HTM outflow physiology and the response to biological agents. The pore size of the nanofibers and the initial cell concentration influence cell attachment, surface coverage, and monolayer formation. The JCT construct maintains a characteristic HTMC phenotype as demonstrated by the cell morphology, expression of HTM markers, ECM secretion, and outflow resistance measurements. The *in vitro* perfusion studies demonstrate that the JCT scaffolds are responsive to Dex and Net treatments and confirm the central role of F-actin filaments and focal adhesions when maintaining the cell morphology and resistance to the outflow. This study opens a way to produce JCT functional scaffolds to improve the understanding between the structural and functional relationship in glaucoma and AH outflow.

## Supporting Information Available

Custom-made solution electrospinning setup, schematic representation of the perfusion system, SEM images of the 10% PCL electrospun nanofibrous scaffold, cell area coverage in percentage after the MTT assay (7 days of culture), *z*-stack to see cell infiltration within the scaffold, cell coverage area after drug treatment with dexamethasone and netarsudil, exemplary image processing used for nucleus aspect ratio quantification, and exemplary confocal image to show the nucleus distribution (cell amount) through the scaffold (PDF)
Video showing the cell infiltration within the scaffold (AVI)

## Supplementary Material

ab3c01071_si_001.pdf

ab3c01071_si_003.avi

## ABBREVIATIONS

AH: aqueous humor
Dex: dexamethasone
ECM: extracellular matrix
HTM: human trabecular meshwork
HTMC: human trabecular meshwork cell
JCT: juxtacanalicular
Net: netarsudil
PCL: polycaprolactone
qPCR: quantitative polymerase chain reaction
SEM: scanning electron microscope
TM: trabecular meshwork
