# Supplementary material for: Nanofibrous PCL-Based Human Trabecular Meshwork for Aqueous Humor Outflow Studies
Source: ACS Biomater Sci Eng. 2023 Sep 19;9(11):6333–44. doi: 10.1021/acsbiomaterials.3c01071 (PMC10646841; doi:10.1021/acsbiomaterials.3c01071)
Supplement: Supplementary file 1 — ab3c01071_si_001.pdf [file ab3c01071_si_001.pdf]

Supporting Information

Nanofibrous PCL-based human trabecular meshwork for aqueous humor outflow studies

*Maria Bikuna-Izagirre, Javier Aldazabal, Leire Extramiana, Javier Moreno-Montañes, Elena Carnero, Jacobo Paredes**

E-mail: jparedes@unav.es; Jacobo Paredes

Number of pages:6

Number of figures: 8

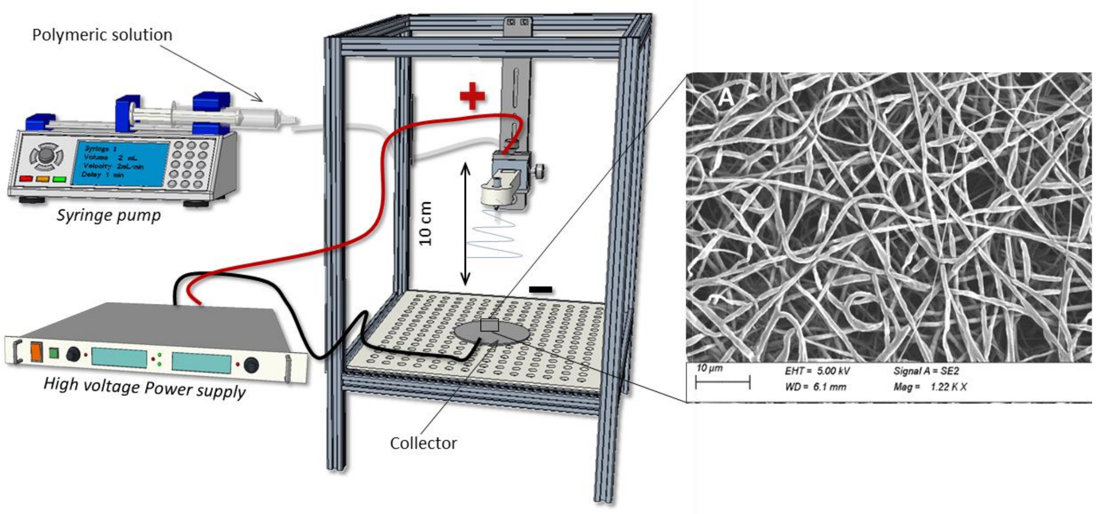

Figure S1: custom-made solution electrospinning set up. (A) PCL 10 % nanofibers. Fabrication parameters: 10 cm, 10 kV, 0.5 mL/h, 20G needle

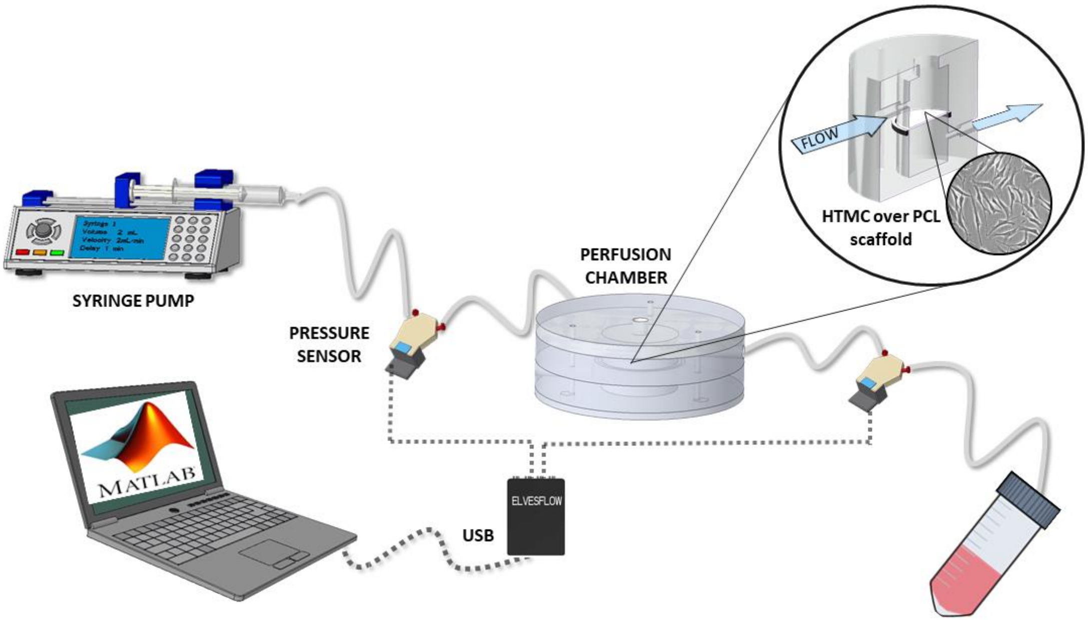

Figure S2: schematic representation of the perfusion system. The system is assembled by a syringe pump (Chemyx Fusion 100), microfluidic pressure sensors (Elveflow, Paris, France) with a pressure range of -70 mBar to 70 mBar, a sensor reader (Elveflow) specific for these pressure sensors, which contains 4 channels, with an acquisition resolution from 9 to 16 bits, and a resolution of 5 mV. An ad hoc designed perfusion chamber, where the PCL nanofibrous membrane (with or without cells) is placed for further perfusion studies. The perfused media (cell media and media plus drugs) is stored as waste in a Falcon tube. The perfusion chamber is placed inside the incubator (at 37 °C and 5 % CO_2) for perfusion studies.

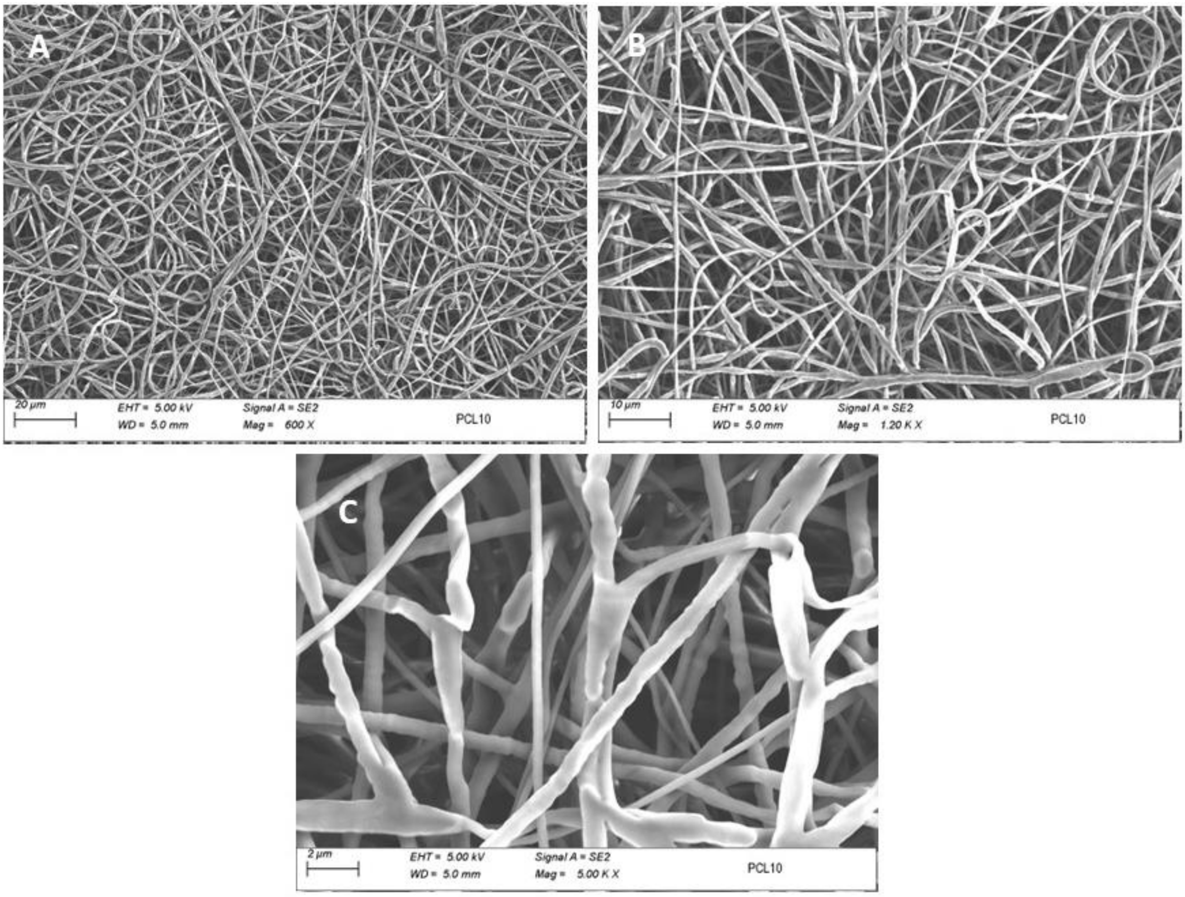

Figure S3: SEM image of PCL 10% electrospun nanofibrous scaffold. (A) Magnification of 600x. Scale bar: 20 µm. (B) Magnification of 1.20Kx. Scale bar: 10 µm. (C) Magnification of 5.00Kx. Scale bar: 2 µm.

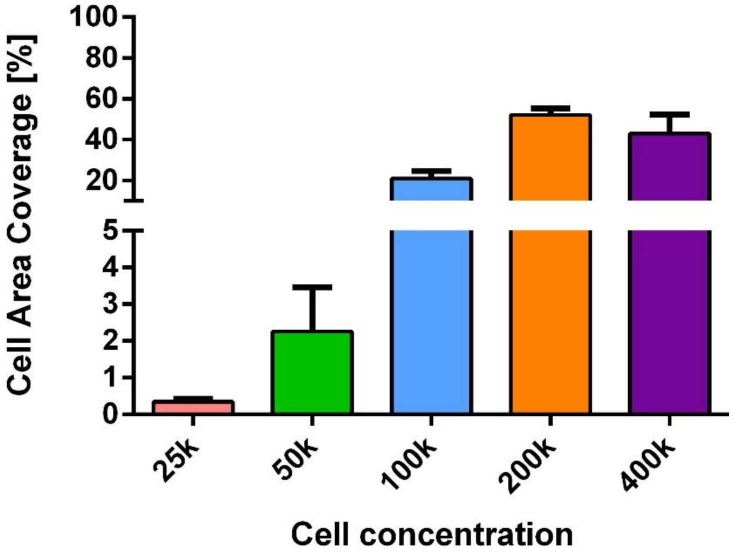

Figure S4: cell area coverage in percentage after MTT assay (7 days of culture).

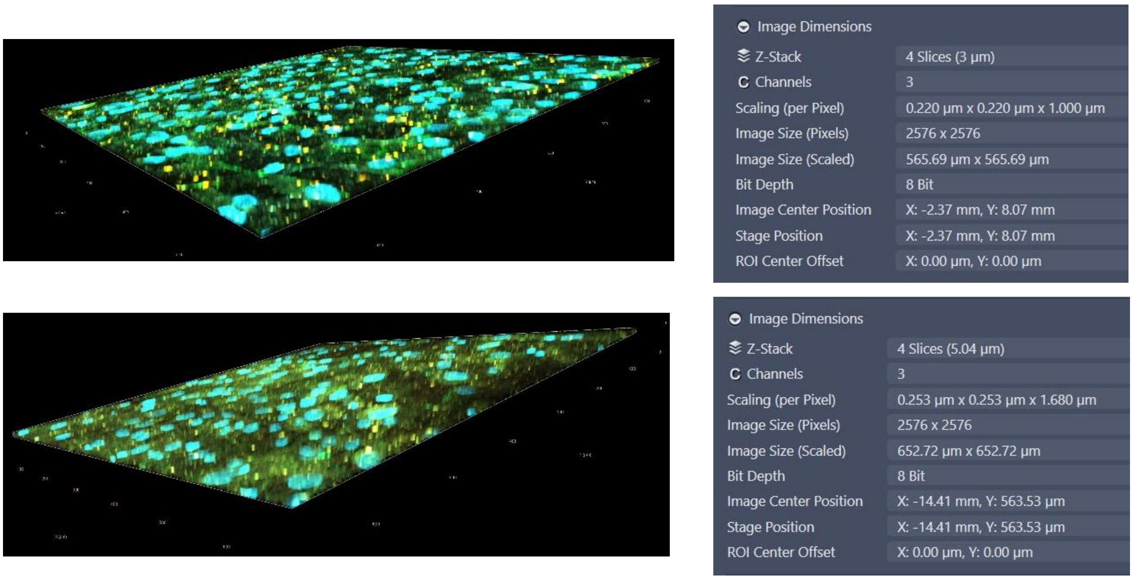

Figure S5: z-stack to see cell infiltration within the scaffold.

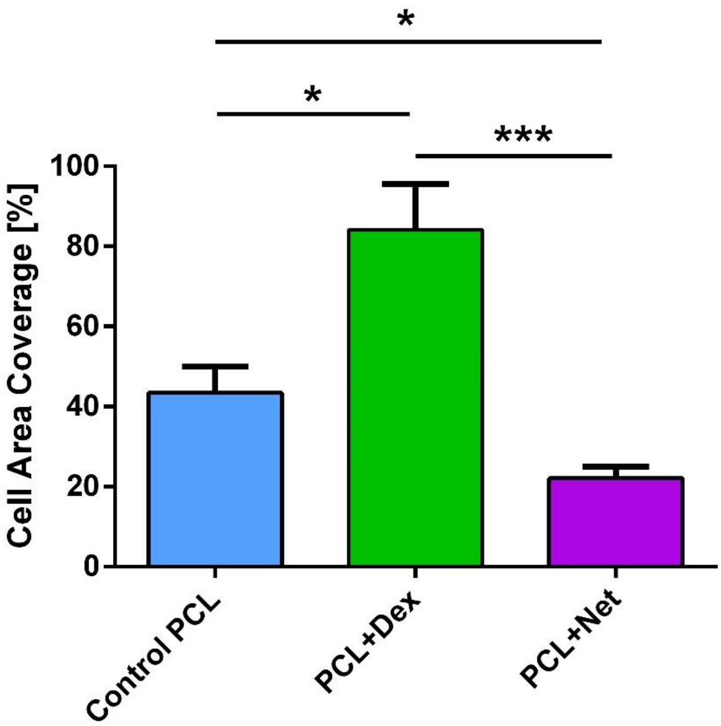

Figure S6: cell coverage area after drug treatment with dexamethasone and Netarsudil. Areas percentages were calculated from SEM images of Figure 3.

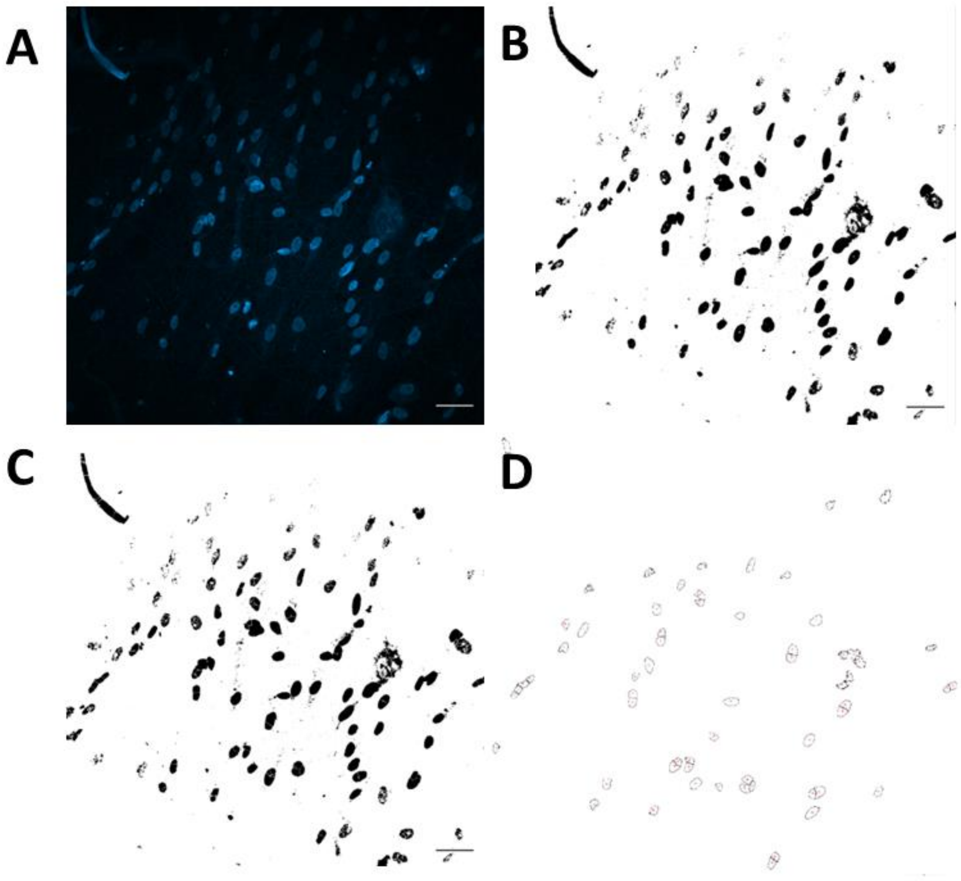

Figure S7: Exemplary image processing used for nuclei aspect ratio quantification. (A) Original nuclei image stained with DAPI. (B) Image made binary. (C) Image after applying "watershed" function. (D) Nuclei considered in the calculation after applying size exclusion limit (50-150 μm). Note that the nuclei in case of clamps of cells or overlapping were excluded from the calculation.

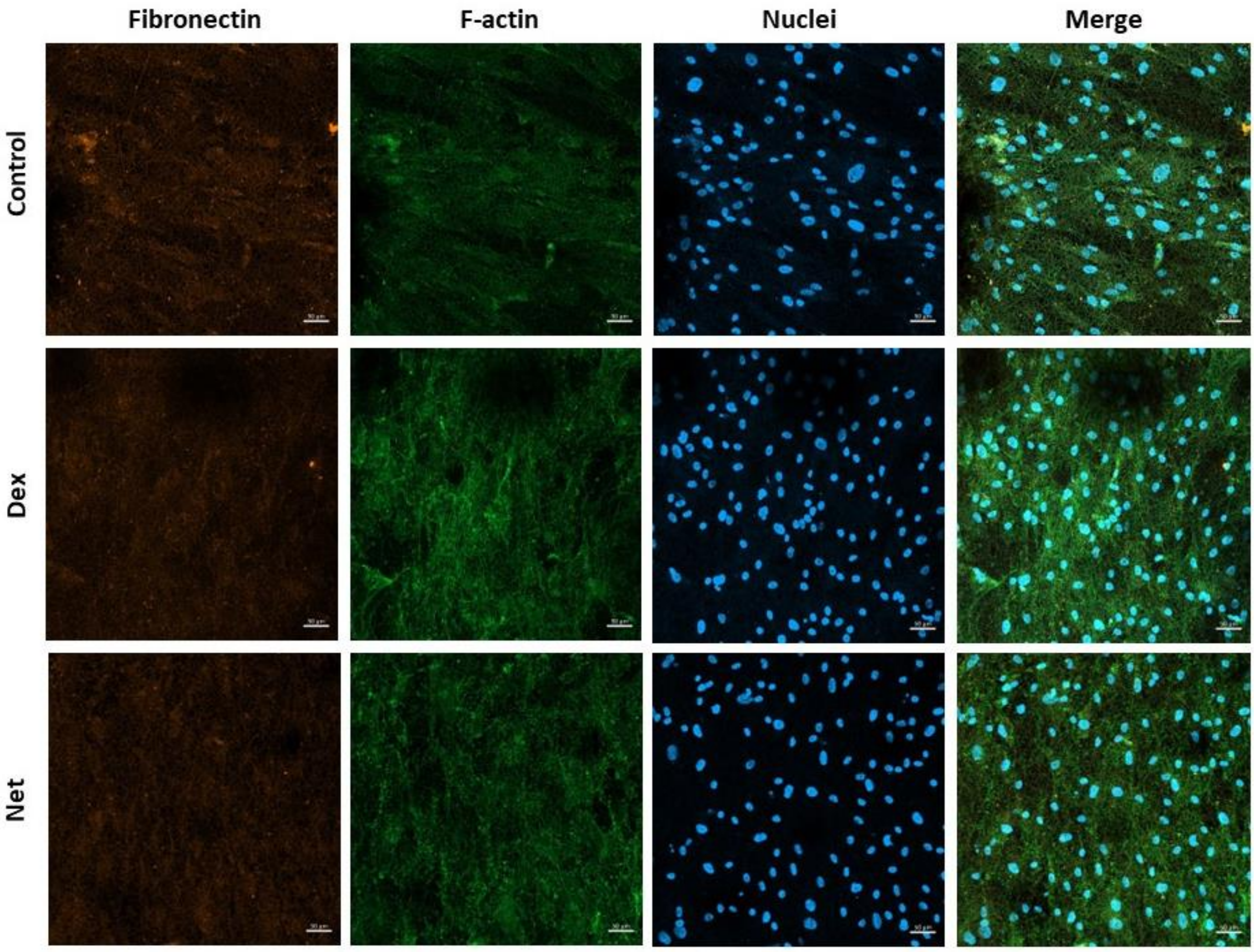

Figure S8: Exemplary confocal image to show the nuclei distribution (cell amount) through the scaffold.
